# Accountability, research transparency and data reporting

**DOI:** 10.1186/s12871-020-01107-6

**Published:** 2020-08-14

**Authors:** Marc Licker, John Diaper, Christoph Ellenberger

**Affiliations:** 1 Department of Anesthesiology, Pharmacology and Intensive Care, University Hospital of Geneva, CH-1211 Geneva, Switzerland; 2 Faculty of Medicine, University of Geneva, CH-1211 Geneva, Switzerland

## Abstract

More than one published paper are often derived from analyzing the same cohort of individuals to make full use of the collected information. Preplanned study outcomes are generally mentioned in open databases while exhaustive information on methodological aspects are provided in submitted articles.

## Letter

Our study published in the BMC Anesthesiology [1] has raised comments and criticisms regarding methodological aspects, namely some preplanned study outcomes and statistical analyses.

In this randomized controlled trial, we investigated the clinical and functional impact of the intravenous administration of Glucose-Insulin-Potassium (GIK) in moderate to high risk patients undergoing coronary artery bypass graft surgery (CABGS) and/or aortic valve replacement (AVR). The protocol was approved by the institutional ethics committee in July 2008 and an abbreviated version was registered in an international open database (ClinicalTrials.gov, NCT00788242). The occurrence of postcardiotomy ventricular dysfunction within the first postoperative 48 h, − a surrogate of low cardiac output syndrome-, was considered the primary study endpoint whereas the release of troponin, cardiovascular complications (myocardial infarct, arrhythmias, stroke) and systolic and diastolic ventricular parameters were considered as secondary outcomes. Comprehensive transesophageal echocardiography (TEE) using speckle tracking, two- and three-dimensional echocardiography were performed in most patients although the quality of imaging and availability of echocardiographers yielded some limitations in data reporting.

Given the bulk of data collected over the perioperative period (> 300 items), the main clinical results of this randomized controlled trial were first reported [2] and the functional results were separately reported and focusing on subsets of patients with coronary artery disease and severe aortic stenosis (given the different baseline-characteristics of the TEE parameters) [1, 3]. As stated in the protocol approved by the ethics committee, TEE measurements were pre-specified and the analysis of the functional changes occurring after GIK infusion and following weaning from cardiopulmonary bypass provided the unique opportunity to investigate the inotropic and lusitropic effects induced by GIK administration before aortic cross-clamping as well as the cardioprotective effects related to GIK infusion following the myocardial ischemic period.

Such “slicing” of a huge amount of data is not exceptional in our scientific world and, more than one published paper is often derived from analyzing the same cohort of individuals to make full use of the collected information. In our trial, the preplanned study outcomes were all described in an open database (ClinicalTrials.gov) without giving exhaustive details on technical aspects. However, the preplanned subgroup analysis was mentioned in the protocol approved by the local ethics committee. Clinical trials are most often registered on web-based database using similar general content. For instance, the VISION trial (vascular events in noncardiac surgery patients cohort evaluation study, NCT00512109) described the general context and study outcomes (e.g., major perioperative vascular events, troponin, stroke) whereas the details on planned analysis are provided in the published papers derived from the cohort analysis [4–11].

Regarding statistical analysis, our study could have been underpowered for some TEE parameters. However, when analyzing patients undergoing aorto-coronary bypass grafting, we performed a post-hoc power calculation for LVEF changes over 3 time points and found that 30 patients per group would be required to detect a between-group difference of 5% (assuming a variance of 64 at each measurement, a correlation of 0.7 between the repeated measurements, a power of 0.8, and a type I error of 0.05). The credibility of a subgroup effect could be increased by an interaction test, especially if important findings in the subgroup differ from the general findings (or from results in other subgroups). This was not the case in our study, the effect of GIK on TEE parameters was similar in CABG and AVR patients making effect modification by the cardiac pathology most unlikely.

Adding a figure to illustrate the TEE changes before and after surgery was helpful to ease the understanding by clinicians. The figure was properly proportionated using a zero line, a full scale and standard deviations, and fully reflected the findings. Confidence intervals, *p*-values and standard deviations are linked together. To be consistent with our publication of TEE data in CABG patients [1], we decided to present the data the same way using mean values with standard deviation in the main text and with 95% confidence intervals in the abstract. In case of interest, confidence intervals can easily be calculated from the given data using the following formula: 95%CI = $\overline{x}$ ± 1.96 × *s*/√n, where s = standard deviation, *n =* sample size.

Accepting or rejecting a null-hypothesis using a predefined *p*-value of, for example 0.05, should indeed be discouraged, as a probability of 4.5% (*p* = 0.045) and 5.5% (*p* = 0.055) is virtually the same. However, the p-value itself remains a useful parameter which describes the probability by witch a difference as demonstrated in a study (or a more extreme difference) occurs by chance and therefore quantifies the evidence against the null hypothesis of no difference.

## Data Availability

All data are available upon request to the corresponding author.

## Abbreviations

AVR: Aortic Valve Replacement
CABGS: Coronary Artery Bypass Graft Surgery (CABGS)
TEE: Transesophageal Echocardiography
GIK: Glucose-Insuline-Potassium
